# Utility of First-Trimester Combined Screening Markers for Identifying Pregnancy at Increased Risk of Fetal Chromosomal Abnormalities

**DOI:** 10.7759/cureus.112478

**Published:** 2026-07-11

**Authors:** Rajani Kumawat, Himanshu Sharma, Archana Nimesh, Saket Sinha, Harmeet Kaur, Prakash Kumar, Indu Priya, Gitanjali Goyal

**Affiliations:** 1 Biochemistry, All India Institute of Medical Sciences, Bathinda, Bathinda, IND; 2 Radiodiagnosis, All India Institute of Medical Sciences, Bathinda, Bathinda, IND; 3 Community and Family Medicine, All India Institute of Medical Sciences, Bathinda, Bathinda, IND

**Keywords:** aneuploidy, chromosomal, dual marker, fetal, first trimester, free β-hcg, nipt, nuchal translucency, papp-a, prenatal screening

## Abstract

Background

First-trimester combined screening using biochemical and ultrasonographic markers is widely employed for early identification of pregnancies at increased risk for fetal screening-based high risk status. However, contemporary Indian data evaluating the performance of these screening markers and their follow-up outcomes remain limited.

Objectives

To evaluate the association of maternal clinico-demographic characteristics and first-trimester biochemical and ultrasonographic markers with chromosomal anomaly risk status and to assess follow-up outcomes of high-risk pregnancies.

Methods

This retrospective cross-sectional analytical study included 164 pregnant women who underwent first-trimester aneuploidy screening between 11 and 13+6 weeks of gestation at a tertiary care centre. Maternal demographic characteristics, biochemical markers [free β-human chorionic gonadotropin (β-hCG) and pregnancy-associated plasma protein-A (PAPP-A)], and ultrasonographic parameters [nuchal translucency (NT) and crown-rump length (CRL)] were analysed. Statistical analyses included Fisher's exact test, Fisher-Freeman-Halton exact test, Mann-Whitney U test, Spearman's correlation analysis, and receiver operating characteristic (ROC) curve analysis to evaluate the discriminatory performance of biochemical markers for identifying pregnancies classified as high risk by first-trimester screening.

Results

Of the 164 participants, 10 (6.1%) were classified as high risk for chromosomal anomalies. Maternal clinico-demographic variables showed no significant association with high risk of chromosomal anomaly, except for the gravida category (p=0.026). High-risk pregnancies demonstrated significantly higher free β-hCG levels and free β-hCG multiples of the median (MoM) values and significantly lower PAPP-A and PAPP-A MoM values compared with low-risk pregnancies (all p<0.01). NT thickness also differed significantly between groups (p=0.042), whereas CRL showed no significant difference. ROC analysis demonstrated good predictive performance for free β-hCG MoM (AUC=0.76, p=0.006), while low PAPP-A MoM showed excellent discriminatory performance after inversion of direction [area under the curve (AUC)=0.937], indicating a strong association with screening-based high-risk status. All 10 high-risk pregnancies underwent non-invasive prenatal testing (NIPT); one pregnancy had a high-risk NIPT result that was subsequently confirmed by amniocentesis as a chromosomal anomaly, resulting in medical termination of pregnancy.

Conclusion

Combined first-trimester screening using free β-hCG, PAPP-A, and ultrasonographic assessment effectively identified pregnancies at increased screening risk for fetal chromosomal abnormalities. These findings should be interpreted cautiously because only one chromosomal abnormality was confirmed.

## Introduction

Fetal screening-based high-risk status, particularly trisomy 21 (Down syndrome), trisomy 18 (Edwards syndrome), and trisomy 13 (Patau syndrome), is a major cause of fetal loss, congenital anomalies, and long-term neurodevelopmental disability worldwide [1,2]. Early identification of pregnancies at increased risk for these screening-based high-risk statuses facilitates timely genetic counselling, informed decision-making, confirmatory diagnostic testing, and appropriate antenatal management. Consequently, prenatal screening has become an integral component of routine obstetric care [3].

Combined first-trimester screening performed between 11 and 13+6 weeks of gestation is a well-established approach for assessing fetal aneuploidy risk [4]. This screening strategy integrates maternal age with maternal serum biochemical markers and ultrasonographic parameters to improve detection rates while minimizing unnecessary invasive procedures. Maternal serum free β-human chorionic gonadotropin (free β-hCG) and pregnancy-associated plasma protein-A (PAPP-A) are the principal biochemical markers used in first-trimester screening protocols [5]. Pregnancies affected by trisomy 21 are typically characterized by elevated free β-hCG levels and reduced PAPP-A concentrations, reflecting abnormal placentation and altered trophoblastic function [5,6].

Ultrasonographic markers further enhance screening performance. Increased fetal nuchal translucency (NT) thickness is strongly associated with screening-based high-risk status and certain structural anomalies, particularly congenital cardiac defects [7]. Assessment of additional sonographic markers, such as the fetal nasal bone, has been shown to further improve screening performance and reduce false-positive rates [8]. Large prospective studies have demonstrated that combined first-trimester screening can achieve detection rates approaching 85-90% for trisomy 21 with acceptable false-positive rates [2,4,9].

In recent years, cell-free fetal DNA-based non-invasive prenatal testing (NIPT) has emerged as a highly accurate screening modality for common fetal aneuploidies [3,10]. Although NIPT offers superior sensitivity and specificity compared with conventional screening methods, its universal implementation remains challenging in many low- and middle-income countries because of financial and logistical constraints [10]. Consequently, combined first-trimester biochemical and ultrasonographic screening continues to serve as a practical, accessible, and cost-effective first-line screening strategy in routine antenatal practice. Current international guidelines recommend a contingent screening approach in which pregnancies identified as high risk by first-trimester screening undergo further evaluation using NIPT and, when indicated, confirmatory invasive diagnostic procedures such as chorionic villus sampling (CVS) or amniocentesis [3].

The diagnostic performance of first-trimester screening markers may vary across populations owing to differences in ethnicity, maternal characteristics, nutritional status, body mass index, and healthcare practices [11]. Despite increasing utilization of dual-marker screening in India, limited real-world Indian data are available regarding the integrated performance of biochemical and ultrasonographic markers in routine clinical settings [12]. Population-specific evaluation is important because variability in biochemical marker distributions and maternal characteristics may influence screening interpretation and risk estimation.

Furthermore, most available evidence primarily focuses on the diagnostic performance of screening markers, whereas data regarding the complete follow-up pathway of pregnancies identified as high risk remain limited, particularly in the Indian setting. Information regarding the practical implementation of a contingent screening strategy involving dual-marker screening, subsequent NIPT, confirmatory invasive diagnostic procedures, and pregnancy outcomes is scarce. Such evidence is essential for optimizing prenatal screening pathways and improving antenatal care delivery in resource-constrained healthcare systems.

The present study addresses this important knowledge gap by providing contemporary real-world evidence from an Indian tertiary care centre regarding the utility of first-trimester combined screening for fetal chromosomal anomaly risk assessment. In addition to evaluating maternal clinico-demographic characteristics, biochemical markers, and ultrasonographic parameters, the study incorporates follow-up assessment of all high-risk pregnancies through a contingent screening approach involving NIPT and confirmatory invasive testing when indicated.

By integrating maternal factors, biochemical markers, ultrasonographic findings, and follow-up outcomes within a single cohort, the present study provides a comprehensive evaluation of prenatal aneuploidy screening in routine clinical practice. The findings contribute population-specific Indian data and offer practical evidence supporting the continued utility of combined first-trimester screening as an effective and cost-conscious first-line strategy for prenatal risk assessment in settings where universal NIPT implementation may not be feasible.

Therefore, the present study was undertaken to evaluate the association of maternal clinico-demographic characteristics, first-trimester biochemical markers, and ultrasonographic parameters with chromosomal anomaly risk status among pregnant women undergoing routine aneuploidy screening at a tertiary care centre. The study also assessed the utility of combined biochemical and radiological markers and documented follow-up outcomes of pregnancies classified as high risk during first-trimester screening.

## Materials and methods

Study design

This retrospective observational analytical study was conducted using records of pregnant women who underwent first-trimester combined aneuploidy screening (dual-marker test) between April 2024 and April 2026 at a tertiary care centre.

Objectives

Primary Objective

To evaluate the association of maternal demographic, biochemical and ultrasonographic markers with chromosomal anomaly risk status.

Secondary Objectives

To assess the predictive performance of biochemical markers using ROC analysis and to document the follow-up outcomes of pregnancies classified as high risk during first-trimester screening.

Sample size

Since this was a retrospective record-based study, all eligible pregnant women fulfilling the inclusion criteria during the study period were included. A total of 164 participants with complete clinical, biochemical, and ultrasonographic records constituted the final study sample.

Study participants

Inclusion Criteria

Pregnant women who underwent first-trimester combined aneuploidy screening between 11 and 13+6 weeks of gestation during the study period were eligible for inclusion. Only participants with complete maternal demographic, clinical, biochemical, and ultrasonographic records, including free β-hCG, PAPP-A, crown-rump length (CRL), and nuchal translucency (NT) measurements, were included in the final analysis. Only complete records were included; therefore, no missing data imputation was required.

Exclusion Criteria

Pregnancies with incomplete clinical, laboratory, or ultrasonographic data and those involving multiple gestations were excluded to ensure uniformity of risk assessment and accuracy of statistical analysis.

Study setting

The study was conducted in the Department of Biochemistry, All India Institute of Medical Sciences (AIIMS), Bathinda.

Data collection

Maternal demographic and clinical variables included age, body mass index (BMI), gravida status, gestational age, history of vaginal bleeding, HCG administration, thyroid disorders, diabetes mellitus, and hypertension.

Risk assessment

Participants were categorized into low-risk and high-risk groups for aneuploidy based on combined maternal clinico-demographical, biochemical, and ultrasound findings at the time of screening. Risk categorization was performed based on calculated risk estimates for fetal aneuploidies by the Life Cycle software version 7.0 Rev. 5 SP1 (PerkinElmer, Finland). A cut-off <1:250 was considered high risk for Down syndrome (Trisomy 21), while a cut-off of <1:100 was considered high risk for both Edwards syndrome (Trisomy 18) and Patau syndrome (Trisomy 13). The study evaluated screening-based risk categorization rather than confirmed fetal screening-based high-risk status.

High-Risk Pregnancy Follow-up

Follow-up information was obtained from the Department of Obstetrics and Gynaecology records. Outcomes assessed included NIPT performance, NIPT results, invasive diagnostic testing (amniocentesis/CVS), confirmation of screening-based high-risk status, and pregnancy outcome.

Biochemical analysis

Maternal venous blood samples were collected as part of routine first-trimester aneuploidy screening, and serum was separated for analysis of free β-human chorionic gonadotropin (free β-hCG) and pregnancy-associated plasma protein-A (PAPP-A). Quantitative estimation of these biomarkers was performed using the Victor²™ D fluorometer (PerkinElmer 1420 Multilabel Counter, PerkinElmer, Finland), based on time-resolved fluoroimmunometric assay (DELFIA) technology. The internal quality assurance for biochemical analysis and the risk assessment procedure have been included according to the manufacturer. This method utilizes lanthanide chelate-labelled antibodies that generate delayed fluorescence signals, thereby minimizing background interference and providing high analytical sensitivity and specificity. Internal quality control procedures were performed throughout the study period in accordance with the manufacturer's recommendations. The measured concentrations were converted to multiples of the median (MoM) after adjustment for gestational age and relevant maternal characteristics using LifeCycle software version 7.0 Rev. 5 SP1, and these adjusted values were used for risk estimation and statistical analysis.

Ultrasonographic examination

Ultrasonographic examination was performed as part of routine first-trimester screening (11-13+6 weeks of gestation) according to the institutional standard first-trimester screening protocol based on established Fetal Medicine Foundation (FMF) measurement guidelines. Nuchal translucency (NT) and crown-rump length (CRL) measurements were obtained by trained obstetric sonologists using standardized techniques and were entered into the LifeCycle software for calculation of screening-based risk.

Outcome measures

Primary Outcome

High-risk chromosomal anomaly status.

Secondary Outcome

NIPT results, confirmed screening-based high-risk status, and pregnancy outcome.

Statistical analysis

Statistical analysis was performed using IBM SPSS Statistics version 29 (IBM Corp., Armonk, NY, USA). software. Continuous variables were assessed for normality using the Shapiro-Wilk test. Normally distributed variables were expressed as mean ± standard deviation (SD), whereas non-normally distributed variables were presented as median with interquartile range (IQR). Categorical variables were summarised as frequencies and percentages. Comparisons between high-risk and low-risk groups were performed using an independent samples Mann-Whitney U test for continuous non-parametric variables, Fisher’s exact test for 2×2 categorical variables, and Fisher-Freeman-Halton exact test for categorical variables with more than two groups. Correlation between biochemical and ultrasonographic markers was assessed using Spearman’s rank correlation coefficient (ρ). A p-value <0.05 was considered statistically significant. Receiver operating characteristic (ROC) curve analysis was performed to evaluate the discriminatory ability of biochemical markers for identifying pregnancies at high risk for chromosomal anomalies. Area under the curve (AUC) with 95% confidence intervals was calculated.

## Results

A total of 164 pregnant women were included in the study. The mean maternal age was 29.9 ± 3.9 years, and the mean BMI was 23.5 ± 4.2 kg/m². Most participants belonged to the 26-30 years age group (45.7%), followed by 31-35 years (37.2%). The median gestational age at screening was 12 weeks and three days. The median free β-hCG MoM and PAPP-A MoM values were 1.1 (IQR 1.2) and 0.98 (IQR 0.70), respectively. The median NT thickness was 1.1 mm (IQR 0.4). Overall, 10 of 164 participants (6.1%) were classified as high risk for chromosomal anomalies. High-risk status for trisomy 21 was identified in eight women (4.9%), while high-risk status for trisomy 18 and trisomy 13 was observed in two participants (1.2%) each (Table 1).

**Table 1:** Baseline Maternal Clinico-demographic Characteristics of Study Participants. Data are presented as n (%). Comparisons between high-risk and low-risk groups were performed using Fisher’s Exact test or Fisher–Freeman–Halton Exact test, as appropriate. A p-value <0.05 was considered statistically significant. Note: Due to small expected cell frequencies, Fisher's Exact (for 2x2 table) or Fisher-Freeman-Halton Exact test (for other than 2x2 table) was applied. A significant association was observed between gravida category and Overall Risk for Chromosomal Aneuploidy Status. SD: standard deviation; IQR: interquartile range; BMI: body mass index; hCG: human chorionic gonadotropin; SBP: systolic blood pressure; DBP: diastolic blood pressure; HTN: hypertension; CORR MOM: corrected multiple of the median; PAPP-A: pregnancy-associated plasma protein-A; NT: nuchal translucency. *Statistically significant association.

Variable	Category	n (%) / Mean ± SD^*^ / Median (IQR)^#^
Age (Mean ± SD)		29.9 ± 3.9^*^
Age (Years)	20-25	17 (10.4)
	26-30	75 (45.7)
	31-35	61 (37.2)
	>35	11 (6.7)
BMI (Mean ± SD)		23.5 ± 4.2^*^
BMI (kg/m^2^)	Underweight	17 (10.4)
	Normal	60 (36.6)
	Overweight	32 (19.5)
	Obese	55 (33.5)
Gestational Age (Median (IQR)		12wk 3day (1wk)^#^
HCG Injection administered	Yes	4 (2.4)
	No	160 (97.6)
Bleeding	Yes	17 (10.4)
	No	147 (89.6)
Gravida	1	61 (37.2)
	2	55 (33.5)
	3	22 (13.4)
	≥4	26 (15.9)
Thyroid Disorder	Euthyroid	157 (95.7)
	Hyperthyroid	5 (3)
	Hypothyroid	2 (1.2)
Diabetic	Yes	6 (3.7)
	No	158 (96.3)
SBP (mmHg) (Median (IQR)		110 (20)^#^
DBP (mmHg) (Median (IQR)		70 (20)^#^
Hypertension Status	Normotensive	159 (97)
	Mild to Moderate HTN	5 (3)
hCGB (Median (IQR)		37.8 (42.4)
hCGB CORR.MOM (Median (IQR)		1.1 (1.2)
PAPP-A (Median (IQR)		3025 (2640)
PAPP-A CORR.MOM (Median (IQR)		0.98 (0.70)
NT (Median (IQR)		1.1 (0.4)^#^
NT CORR.MOM (Median (IQR)		0.8 (0.3)^#^
T21 Aneuploidy Risk Status	High	8 (4.9)
	Low	156 (95.1)
T18 Aneuploidy Risk Status	High	2 (1.2)
	Low	162 (98.8)
T13 Aneuploidy Risk Status	High	2 (1.2)
	Low	162 (98.8)
Overall Risk for Chromosomal Anomaly Status	High	10 (6.1)
	Low	154 (93.9)

Follow-up outcomes of high-risk pregnancies

**Figure 1:**
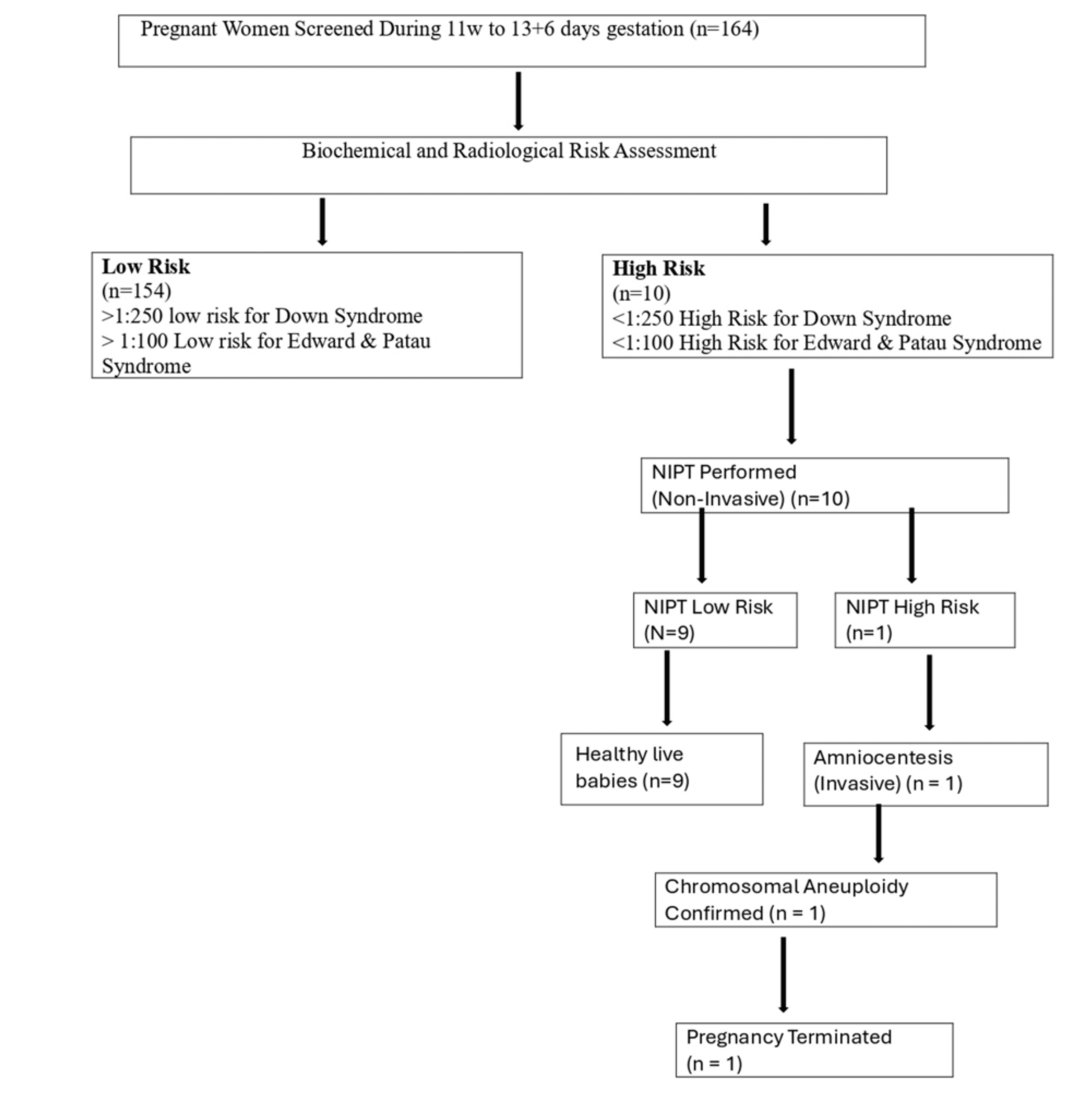
Flow Diagram of Screening Pathway and Follow-up Outcomes of Pregnancies Classified as High Risk for Chromosomal Aneuploidies Flow diagram illustrating participant classification following first-trimester combined screening, subsequent NIPT evaluation, invasive diagnostic confirmation, and pregnancy outcomes among high-risk pregnancies. NIPT: non-invasive prenatal testing.

Follow-up information was available for all pregnancies classified as high risk on first-trimester screening. All 10 high-risk participants underwent non-invasive prenatal testing (NIPT). Among these, one participant (10.0%) had a high-risk NIPT result, whereas nine participants (90.0%) had low-risk NIPT results. The participant with a high-risk NIPT result subsequently underwent amniocentesis, which confirmed a chromosomal abnormality. Following genetic confirmation, the pregnancy was medically terminated. No chromosomal abnormalities were identified among pregnancies classified as low risk in the follow-up (Figure 1). No pregnancy classified as low risk was subsequently found to have a confirmed chromosomal abnormality during the available follow-up period.

Association between clinico-demographic characteristics and chromosomal aneuploidy risk was assessed

No statistically significant association was observed between chromosomal anomaly risk status and maternal age category (p=0.594), BMI category (p=0.706), history of vaginal bleeding (p=1.000), HCG administration (p=1.000), thyroid disorder (p=1.000), diabetes mellitus (p=1.000), or hypertension status (p=1.000). However, gravida category demonstrated a statistically significant association with chromosomal anomaly risk status (p=0.026), with increased risk more frequently observed among women with gravida ≥3 (Table 2).

**Table 2:** Association between Clinico-demographic characteristics and Chromosomal Anomaly Status Data are presented as n (%). Comparisons between high-risk and low-risk groups were performed using Fisher’s Exact test or Fisher–Freeman–Halton Exact test, as appropriate. A p-value <0.05 was considered statistically significant. Note: Due to small expected cell frequencies, Fisher's Exact (for 2x2 table) or Fisher-Freeman-Halton Exact test (for other than 2x2 table) was applied. A significant association was observed between gravida category and Overall Risk for Chromosomal Aneuploidy Status. BMI: body mass index; hCG: human chorionic gonadotropin; HTN: hypertension;  *Statistically significant association.

Variable	Category	Chromosomal Anomaly Risk Status			
		High	Low	p-Value	Statistical Test
Age (Years) Category	20-25	0 (0)	17 (100)	0.594	Fisher-Freeman-Halton Exact Test
	26-30	4 (5.3)	71 (94.7)		
	31-35	5 (8.2)	56 (91.8)		
	>35	1 (9.1)	10 (90.9)		
BMI (kg/m^2^) Category (<18.5:Underweight, 18.5-22.9: Normal, 23-24.9: Overweight ≥25: Obese )		2 (11.8)	15 (88.2)	0.706	Fisher-Freeman-Halton Exact Test
	Normal	3 (5)	57 (95)		
	Overweight	2 (6.3)	30 (93.8)		
	Obese	3 (5.5)	52 (94.5)		
HCG Injection	Yes	0 (0)	4 (100)	1	Fisher's Exact Test
	No	10 (6.3)	150 (93.8)		
Bleeding	Yes	1 (5.9)	16 (94.1)	1	Fisher's Exact Test
	No	9 (6.1)	138 (93.9)		
Gravida Category	1	4 (6.6)	57 (93.4)	0.026*	Fisher-Freeman-Halton Exact Test
	2	0 (0)	55 (100)		
	3	3 (13.6)	19 (86.4)		
	≥4	3 (11.5)	23 (88.5)		
Thyroid Disorder	Hyperthyroid	0 (0)	2 (100)	1	Fisher-Freeman-Halton Exact Test
	Hypothyroid	0 (0)	5 (100)		
	Euthyroid	10 (6.4)	147 (93.6)		
Diabetes Category	Yes	0 (0)	6 (100)	1	Fisher's Exact Test
	No	10 (6.3)	148 (93.7)		
HTN Category (Before 20wk)	Normotensive	10 (6.3)	149 (93.7)	1	Fisher's Exact Test
	Mild to Moderate HTN	0 (0)	5 (100)		

Comparison of biochemical and ultrasonographic markers between high-risk and low-risk groups was studied

Women classified as high risk for chromosomal anomalies demonstrated significantly altered biochemical marker profiles compared with the low-risk group. Median free β-hCG concentrations were significantly higher among high-risk pregnancies [126.55 (IQR 81.45) vs. 36.27 (IQR 36.66); p=0.007]. Similarly, free β-hCG MoM values were significantly elevated in the high-risk group [2.51 (IQR 2.50) vs. 1.06 (IQR 0.85); p=0.006]. In contrast, median PAPP-A concentrations [815 (IQR 2365) vs. 3140 (IQR 2780); p<0.001] and PAPP-A MoM values [0.268 (IQR 0.46) vs. 1.025 (IQR 0.69); p<0.001] were significantly lower among high-risk pregnancies. NT thickness showed a modest but statistically significant difference between groups (p=0.042), whereas NT MoM and CRL did not differ significantly (Table 3).

**Table 3:** Comparison of Biochemical Markers and Ultrasonographic Parameters Between High-Risk and Low-Risk T21 Groups Data are presented as median interquartile range (IQR). Comparisons were performed using the Independent-Samples Mann–Whitney U test. A p-value <0.05 was considered statistically significant. CRL: crown-rump length; hCGβ: free beta-human chorionic gonadotropin; PAPP-A: pregnancy-associated plasma protein-A; CORR.MOM: corrected multiples of median; NT: nuchal translucency. # Independent-Samples Mann-Whitney U Test. *Statistically significant difference between groups.

	Overall Risk for Chromosomal Aneuploidy risk Status in T21 group’s		
	High	Low	p-Value#
	Median (IQR)	Median (IQR)	
CRL(mm)	64.17 (18.1)	59.8 (11.1)	0.839
hCGB	126.55 (81.45)	36.27 (36.66)	0.007*
hCGB CORR.MOM	2.51 (2.5)	1.06 (0.85)	0.006*
PAPP-A	815 (2365)	3140 (2780)	<0.001*
PAPP-A CORR.MOM	0.268 (0.46)	1.025 (0.69)	<0.001*
NT	0.95 (0.27)	1.14 (0.4)	0.042*
NT CORR.MOM	0.63 (0.38)	0.78 (0.31)	0.057

Correlation analysis

Spearman's correlation analysis demonstrated weak correlations between biochemical and ultrasonographic markers. NT thickness showed a weak negative correlation with free β-hCG MoM (ρ=−0.138, p=0.078) and PAPP-A MoM (ρ=−0.068, p=0.386). A weak positive correlation was observed between free β-hCG MoM and PAPP-A MoM (ρ=0.124, p=0.114). None of these associations reached statistical significance (Table 4). The multivariable adjustment was not performed because of the very small number of high-risk cases, which precluded reliable multivariable modelling.

**Table 4:** Correlation Between Biochemical and Ultrasonographic Markers (Spearman’s Correlation Analysis) Spearman’s rank correlation coefficient (ρ) was used to evaluate the relationship between biochemical and ultrasonographic markers. Values are presented as Spearman’s rho (ρ) and p-values. A p-value <0.05 was considered statistically significant. NT: nuchal translucency; hCGβ: free beta-human chorionic gonadotropin; PAPP-A: pregnancy-associated plasma protein-A; MoM: multiples of median.

Variables Compared	Spearman’s Correlation Coefficient (ρ)	p-value	Interpretation
NT vs Free β-hCG MoM	-0.138	0.078	Weak negative correlation, not statistically significant
NT vs PAPP-A MoM	-0.068	0.386	Very weak negative correlation, not statistically significant
PAPP-A MoM vs Free β-hCG MoM	0.124	0.114	Weak positive correlation, not statistically significant

ROC analysis

**Figure 2:**
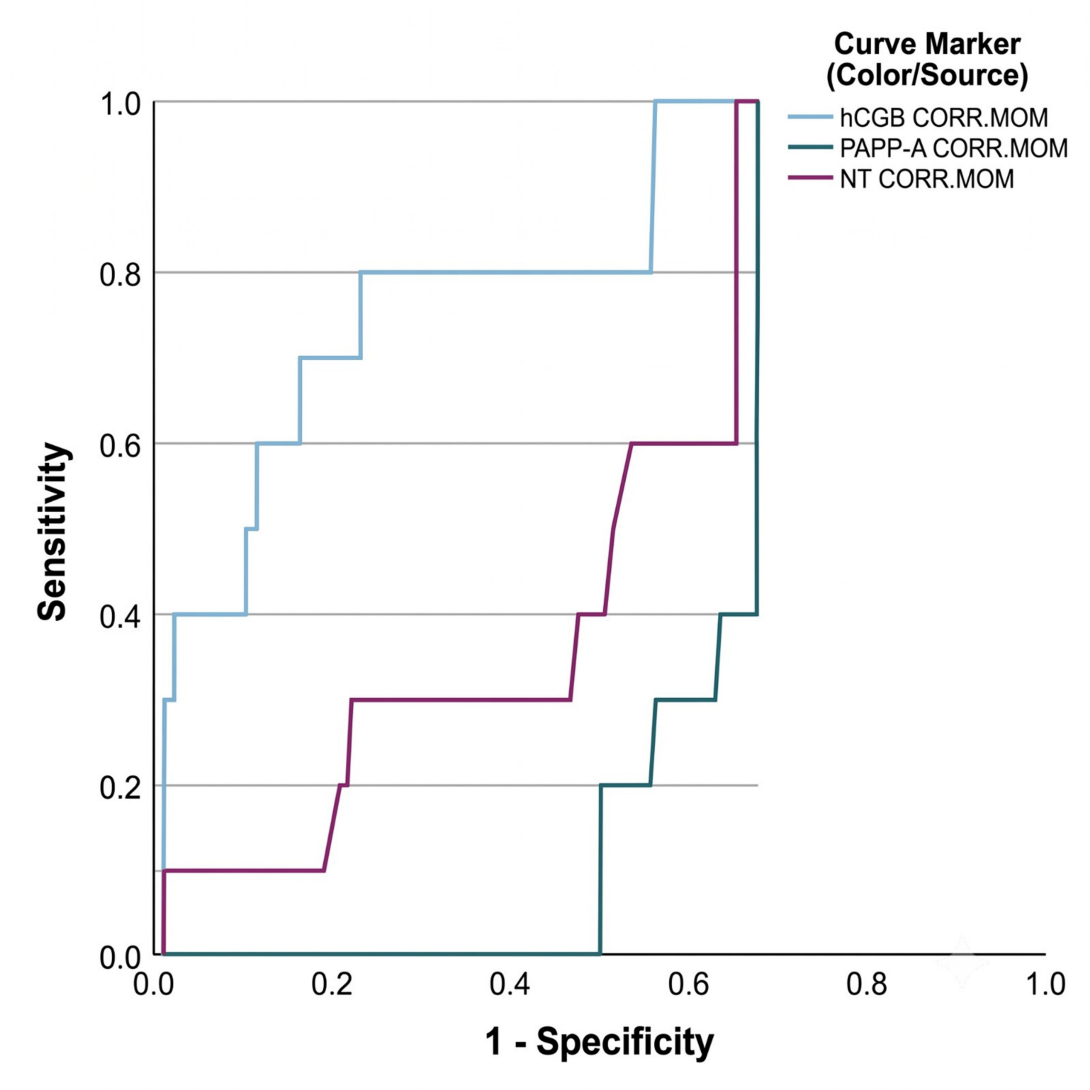
ROC Curve Analysis for Predictive Performance of Biochemical Markers for Chromosomal Anomaly Status ROC curves demonstrating the discriminatory performance of free β-hCG MoM, PAPP-A MoM, and NT MoM for identifying pregnancies classified as high risk for chromosomal aneuploidies. AUC: area under the curve; ROC: receiver operating characteristic; MoM: multiples of median; NT: nuchal translucency.

ROC analysis demonstrated that Free β-hCG MoM had fair-to-good diagnostic accuracy for predicting chromosomal anomaly status (AUC=0.76, p=0.006). NT MoM showed poor and non-significant discriminatory performance (AUC=0.32, p=0.057). PAPP-A MoM demonstrated a highly significant inverse discriminatory relationship (AUC=0.073, p<0.001), suggesting that lower PAPP-A values are strongly associated with screening-based high-risk status and may provide excellent predictive utility when interpreted in the appropriate direction (Table 5, Figure 2).

**Table 5:** Diagnostic Performance of First-Trimester Ultrasonographic and Biochemical Markers for Predicting High Risk of Chromosomal Anomalies: ROC Curve Analysis The AUC below 0.5 reflects the inverse relationship between PAPP-A MoM levels and chromosomal anomaly risk. Lower PAPP-A MoM concentrations were associated with increased risk; therefore, reversing the direction of the marker would yield an AUC greater than 0.90.

Marker	AUC (95% CI)	Standard Error	p-value	Interpretation
NT MoM	0.358 (0.072–0.645)	0.146	0.241	Poor discriminatory performance; not statistically significant
Free β-hCG MoM	0.804 (0.565–1.000)	0.122	0.012	Good predictive performance; statistically significant
PAPP-A MoM	0.079 (0.000–0.164)	0.043	<0.001	Inverse discriminatory relationship with outcome

## Discussion

First-trimester combined screening using maternal serum biochemical markers and ultrasonographic parameters remains the cornerstone of prenatal risk assessment for fetal screening-based high-risk status. In the present study, 10 of 164 screened pregnancies (6.1%) were classified as high risk for chromosomal anomalies. High-risk pregnancies demonstrated significantly elevated free β-hCG and free β-hCG MoM values together with significantly reduced PAPP-A and PAPP-A MoM values compared with low-risk pregnancies. Furthermore, follow-up evaluation revealed that all high-risk pregnancies underwent NIPT, and one case was subsequently confirmed as a chromosomal abnormality by amniocentesis.

Maternal age is traditionally regarded as one of the strongest risk factors for fetal aneuploidies and forms an integral component of all prenatal screening algorithms [1,3]. However, no significant association was observed between maternal age categories and chromosomal anomaly risk status in the present study. Similar findings have been reported by Malone et al. and Kagan et al., who demonstrated that incorporation of biochemical and ultrasonographic markers significantly improves risk prediction beyond maternal age alone [2,4]. The absence of a significant association in our study may be attributable to the relatively small number of high-risk pregnancies and the concentration of participants within a relatively narrow reproductive age range. These findings support the growing consensus that maternal age alone is insufficient for accurate risk stratification and should be interpreted in conjunction with objective biochemical and ultrasonographic markers.

Interestingly, gravida status showed a significant association with chromosomal anomaly risk, with higher risk observed among women with gravida ≥3. Although gravida is not routinely included in aneuploidy risk prediction models, this observation may reflect cumulative reproductive factors or the tendency for higher gravidity to coexist with increasing maternal age. Similar associations have occasionally been reported in epidemiological studies; however, evidence remains inconsistent and further investigation in larger cohorts is warranted. The observed association between gravida and screening risk should be interpreted cautiously because of the limited number of high-risk pregnancies and requires confirmation in larger prospective studies.

One of the most important findings of the present study was the significant elevation of free β-HCG and free β-hCG MoM values among high-risk pregnancies. Elevated maternal serum free β-hCG is a characteristic biochemical feature of trisomy 21 pregnancies and reflects altered trophoblastic differentiation and placental function [5]. Spencer et al. first demonstrated that combining free β-hCG with PAPP-A and NT measurement significantly improves detection rates for trisomy 21 during the first trimester [5]. Subsequently, Kagan et al. reported that free β-hCG contributes substantially to the predictive accuracy of combined screening algorithms [4]. Similar observations have been reported in the FASTER trial and the SURUSS study, both of which identified elevated free β-hCG as a key marker associated with fetal aneuploidies [6,9]. The significantly higher free β-hCG MoM values observed in the present study therefore corroborate the established biochemical profile of high-risk pregnancies and reinforce its utility as a major component of first-trimester screening.

A marked reduction in PAPP-A and PAPP-A MoM values was also observed among high-risk pregnancies. Low maternal serum PAPP-A concentrations are among the most consistently reported biochemical abnormalities associated with trisomy 21 and other chromosomal disorders [5,6]. PAPP-A is produced by placental trophoblasts and plays an important role in insulin-like growth factor regulation, placental development, and fetal growth. Reduced levels are thought to reflect impaired trophoblastic invasion and abnormal placentation. In addition to fetal aneuploidies, low PAPP-A concentrations have been associated with adverse pregnancy outcomes including fetal growth restriction, preeclampsia, preterm birth, and stillbirth [13,14]. The present findings are therefore biologically plausible and consistent with previous reports. Importantly, ROC analysis demonstrated strong discriminatory performance of PAPP-A MoM values, suggesting that PAPP-A may represent one of the most informative biochemical markers for early antenatal risk stratification.

The present study also demonstrated significant differences in NT thickness between high-risk and low-risk pregnancies. Increased NT thickness is a well-established marker for screening-based high-risk status, congenital cardiac defects, and several genetic syndromes [7]. Nicolaides and colleagues demonstrated that NT measurement substantially improves detection rates for trisomy 21 when combined with maternal serum markers [7]. The landmark serum, urine and ultrasound screening study (SURUSS) and subsequent investigations confirmed that NT measurement significantly enhances screening sensitivity while maintaining acceptable false-positive rates [9]. Although NT MoM did not achieve statistical significance in the present study, the observed difference in absolute NT values suggests that sonographic assessment continues to provide valuable complementary information for risk prediction. The lack of significance for NT MoM may be attributable to the small number of high-risk pregnancies and the relatively narrow distribution of NT values within the study population.

Correlation analysis demonstrated weak and statistically non-significant associations between biochemical and ultrasonographic markers. Similar findings have been reported by Kagan et al. and Wright et al., who suggested that maternal serum biomarkers and sonographic markers reflect distinct biological pathways involved in fetal and placental development [4,11]. The relative independence of these markers is advantageous because it allows each parameter to contribute unique information to screening algorithms, thereby improving overall predictive performance when used in combination.

A notable strength of the present study is the inclusion of follow-up outcomes for all pregnancies classified as high risk. While many studies terminate their evaluation at the screening stage, the present study assessed the subsequent clinical pathway, including NIPT, invasive diagnostic testing, and pregnancy outcome. All ten high-risk pregnancies underwent NIPT, and one pregnancy demonstrated a high-risk NIPT result that was subsequently confirmed by amniocentesis. This finding supports current international recommendations advocating a contingent screening strategy, whereby pregnancies identified as high risk on first-trimester screening undergo secondary evaluation with cell-free DNA testing before invasive procedures are considered [3,10,15].

The incorporation of follow-up data represents an important contribution to the existing literature because real-world evidence regarding implementation of contingent screening pathways remains limited in India. Contemporary guidelines recommend NIPT as a highly accurate secondary screening modality; however, positive NIPT results still require confirmation through invasive diagnostic procedures owing to the possibility of false-positive results arising from confined placental mosaicism, maternal screening-based high-risk status, or technical factors [3,10,15]. The successful identification and confirmation of a chromosomal abnormality in our study demonstrates the practical utility of combining conventional screening, NIPT, and confirmatory invasive testing within a structured prenatal screening framework.

From a public health perspective, the findings of the present study have particular relevance for low- and middle-income countries. Although NIPT has transformed prenatal screening because of its excellent sensitivity and specificity, universal implementation remains challenging because of cost and accessibility constraints [10,15]. In such settings, combined first-trimester screening using free β-hCG, PAPP-A, and ultrasonographic assessment continues to represent a practical and cost-effective first-line strategy. The present findings support the use of a contingent screening approach that reserves NIPT and invasive testing for pregnancies identified as high risk, thereby optimizing resource utilization while maintaining high-quality prenatal care.

The findings of the present study are also consistent with evolving recommendations and experiences from the Indian prenatal screening landscape. Indian experts have emphasized that combined first-trimester screening using maternal serum biochemical markers and ultrasonographic assessment should continue to serve as the primary screening strategy for fetal screening-based high-risk status, particularly in resource-constrained settings where universal implementation of NIPT may not be feasible. Recent Indian recommendations have supported a contingent screening model in which pregnancies identified as high risk on first-trimester screening undergo further evaluation with NIPT before invasive diagnostic procedures are considered. Such an approach improves risk stratification, reduces unnecessary invasive testing, and optimizes healthcare resource utilization while maintaining high diagnostic accuracy. The complete follow-up pathway observed in the present study, including first-trimester screening, NIPT evaluation, confirmatory amniocentesis, and final pregnancy outcome, further supports the practical applicability of this strategy in routine clinical practice in India [16-18].

The importance of NT measurement in first-trimester screening continues to be emphasized in contemporary obstetric practice. Although NT MoM did not achieve statistical significance in the present study, NT thickness demonstrated a significant difference between high-risk and low-risk groups. Similar observations have been reported in clinical screening programmes where NT serves as an important complementary marker rather than an isolated predictor. The integration of NT assessment with biochemical markers improves overall screening performance and remains an essential component of contemporary first-trimester risk assessment protocols [18].

Finally, the present study contributes valuable population-specific data from India, where evidence regarding integrated prenatal screening pathways remains limited [12]. By incorporating maternal characteristics, biochemical markers, ultrasonographic findings, ROC analysis, and follow-up outcomes within a single cohort, the study provides a comprehensive evaluation of prenatal aneuploidy screening in routine clinical practice. These findings may assist clinicians, policymakers, and public health authorities in refining prenatal screening strategies and improving antenatal care delivery in similar healthcare environments.

Clinical implication

The findings of the present study support the continued use of combined first-trimester screening using free β-hCG, PAPP-A, and ultrasonographic assessment for identifying pregnancies at increased screening risk for fetal chromosomal abnormalities. In resource-constrained settings where universal access to non-invasive prenatal testing (NIPT) may not be feasible, a contingent screening strategy, in which pregnancies identified as high risk undergo further evaluation with NIPT followed by confirmatory invasive testing when indicated, may facilitate appropriate prenatal risk assessment. Although the present findings provide real-world evidence supporting this approach, they should be interpreted in light of the retrospective design and the limited number of confirmed chromosomal abnormalities.

Strengths and limitations

Strengths

The study provides real-world, population-specific data from an Indian tertiary care centre on first-trimester combined screening in routine clinical practice. It evaluated biochemical and ultrasonographic markers using standardized MoM values and included complete follow-up of all pregnancies classified as high risk, documenting NIPT, confirmatory invasive testing when indicated, and pregnancy outcomes. ROC analysis further assessed the discriminatory performance of screening markers for screening-based high-risk status, supporting the feasibility of a contingent screening approach in a resource-limited setting.

Limitations

The retrospective single-centre design and the small number of high-risk pregnancies (n = 10), with only one confirmed chromosomal abnormality, limit the statistical power, generalizability, and diagnostic inference of the findings. As the evaluated markers were also components of the screening algorithm, incorporation (circular) bias may have overestimated their discriminatory performance. Multivariable analysis was not performed because of the limited number of high-risk cases. Confirmatory diagnostic testing was not available for all pregnancies, and long-term follow-up for structural anomalies and neonatal outcomes was unavailable.

Novelty of the Study: The present study provides real-world Indian evidence on the integrated use of first-trimester biochemical and ultrasonographic screening markers and uniquely reports complete follow-up outcomes of all high-risk pregnancies through a contingent screening pathway involving NIPT, confirmatory invasive testing, and pregnancy outcome assessment. This comprehensive evaluation extends beyond conventional screening performance studies and provides practical evidence relevant to resource-limited healthcare settings.

## Conclusions

Combined first-trimester screening using maternal serum free β-hCG, PAPP-A, and ultrasonographic assessment was associated with screening-based high-risk status for fetal chromosomal abnormalities in this cohort. Elevated free β-hCG MoM and reduced PAPP-A MoM were the biochemical markers most strongly associated with high-risk screening status, while ultrasonographic assessment provided complementary information for risk stratification. Follow-up of high-risk pregnancies demonstrated the feasibility of a contingent screening pathway incorporating NIPT and confirmatory invasive testing when indicated. Given the small number of high-risk pregnancies and the single confirmed chromosomal abnormality, these findings should be interpreted cautiously. Larger prospective multicentre studies with comprehensive diagnostic follow-up are warranted to further validate these observations.

## References

[1] Nicolaides KH (1994). Screening for fetal chromosomal abnormalities: need to change the rules. Ultrasound Obstet Gynecol.

[2] Malone FD, Canick JA, Ball RH (2005). First-trimester or second-trimester screening, or both, for Down's syndrome. N Engl J Med.

[3] American College of Obstetricians and Gynecologists’ Committee on Practice Bulletins—Obstetrics, Committee on Genetics (2020). Screening for fetal chromosomal abnormalities: ACOG Practice Bulletin, Number 226. Obstet Gynecol.

[4] Kagan KO, Wright D, Baker A, Sahota D, Nicolaides KH (2008). Screening for trisomy 21 by maternal age, fetal nuchal translucency thickness, free beta-human chorionic gonadotropin and pregnancy-associated plasma protein-A. Ultrasound Obstet Gynecol.

[5] Spencer K, Souter V, Tul N, Snijders R, Nicolaides KH (1999). A screening program for trisomy 21 at 10-14 weeks using fetal nuchal translucency, maternal serum free beta-human chorionic gonadotropin and pregnancy-associated plasma protein-A. Ultrasound Obstet Gynecol.

[6] Dugoff L, Hobbins JC, Malone FD (2004). First-trimester maternal serum PAPP-A and free-beta subunit human chorionic gonadotropin concentrations and nuchal translucency are associated with obstetric complications: a population-based screening study (the FASTER Trial). Am J Obstet Gynecol.

[7] Nicolaides KH (2004). Nuchal translucency and other first-trimester sonographic markers of chromosomal abnormalities. Am J Obstet Gynecol.

[8] Cicero S, Curcio P, Papageorghiou A, Sonek J, Nicolaides K (2001). Absence of nasal bone in fetuses with trisomy 21 at 11-14 weeks of gestation: an observational study. Lancet.

[9] Wald NJ, Rodeck C, Hackshaw AK, Walters J, Chitty L, Mackinson AM (2003). First and second trimester antenatal screening for Down's syndrome: the results of the serum, urine and ultrasound screening study (SURUSS). Health Technol Assess.

[10] Gil MM, Accurti V, Santacruz B, Plana MN, Nicolaides KH (2017). Analysis of cell-free DNA in maternal blood in screening for aneuploidies: updated meta-analysis. Ultrasound Obstet Gynecol.

[11] Wright D, Kagan KO, Molina FS, Gazzoni A, Nicolaides KH (2008). A mixture model of nuchal translucency thickness in screening for chromosomal defects. Ultrasound Obstet Gynecol.

[12] Shiefa S, Amargandhi M, Bhupendra J, Moulali S, Kristine T (2013). First trimester maternal serum screening using biochemical markers Papp-a and free β-hCG for Down syndrome, Patau syndrome and Edward syndrome. Indian J Clin Biochem.

[13] Krantz D, Goetzl L, Simpson JL (2004). Association of extreme first-trimester free human chorionic gonadotropin-beta, pregnancy-associated plasma protein A, and nuchal translucency with intrauterine growth restriction and other adverse pregnancy outcomes. Am J Obstet Gynecol.

[14] Smith GC, Stenhouse EJ, Crossley JA, Aitken DA, Cameron AD, Connor JM (2002). Early pregnancy levels of pregnancy-associated plasma protein a and the risk of intrauterine growth restriction, premature birth, preeclampsia, and stillbirth. J Clin Endocrinol Metab.

[15] Audibert F, Wou K, Okun N, De Bie I, Wilson RD (2024). Prenatal screening for fetal chromosomal anomalies. J Obstet Gynaecol Can.

[16] Phadke SR, Puri RD, Ranganath P (2017). Prenatal screening for genetic disorders: suggested guidelines for the Indian scenario. Indian J Med Res.

[17] Jayashankar SS, Nasaruddin ML, Hassan MF, Dasrilsyah RA, Shafiee MN, Ismail NA, Alias E (2023). Non-invasive prenatal testing (NIPT): reliability, challenges, and future directions. Diagnostics (Basel).

[18] Manikandan K, Seshadri S (2017). Down syndrome screening in India: are we there yet?. J Obstet Gynaecol India.

